# The pseudokinase CaMKv is required for the activity-dependent maintenance of dendritic spines

**DOI:** 10.1038/ncomms13282

**Published:** 2016-10-31

**Authors:** Zhuoyi Liang, Yi Zhan, Yang Shen, Catherine C. L. Wong, John R. Yates, Florian Plattner, Kwok-On Lai, Nancy Y. Ip

**Affiliations:** 1 Division of Life Science, The Hong Kong University of Science and Technology, Hong Kong, China; 2 Molecular Neuroscience Center, The Hong Kong University of Science and Technology, Hong Kong, China; 3 State Key Laboratory of Molecular Neuroscience, The Hong Kong University of Science and Technology, Hong Kong, China; 4 Department of Chemical Physiology, The Scripps Research Institute, La Jolla, California 92037, USA; 5 Department of Psychiatry, University of Texas Southwestern Medical Center, Dallas, Texas 75390–9070, USA

## Abstract

Dendritic spine stabilization depends on afferent synaptic input and requires changes in actin cytoskeleton dynamics and protein synthesis. However, the underlying molecular mechanism remains unclear. Here we report the identification of ‘calmodulin kinase-like vesicle-associated' (CaMKv), a pseudokinase of the CaMK family with unknown function, as a synaptic protein crucial for dendritic spine maintenance. CaMKv mRNA localizes at dendrites, and its protein synthesis is regulated by neuronal activity. CaMKv function is inhibited upon phosphorylation by cyclin-dependent kinase 5 (Cdk5) at Thr345. Furthermore, CaMKv knockdown in mouse hippocampal CA1 pyramidal neurons impairs synaptic transmission and plasticity *in vivo*, resulting in hyperactivity and spatial memory impairment. These findings collectively indicate that the precise regulation of CaMKv through activity-dependent synthesis and post-translational phosphorylation is critical for dendritic spine maintenance, revealing an unusual signalling pathway in the regulation of synaptic transmission and brain function that involves a pseudokinase.

## Abstract (web summary)

CaMKv is a pseduokinase of unknown function. Here, the authors identify the protein as a substrate of the protein kinase Cdk5, and show that CaMKv is synthesized in response to neural activity and plays an important role in maintaining dendritic spines, synaptic plasticity, and hippocampal memory via RhoA inhibition.

The dendritic spines of excitatory synapses constitute individual biochemical and electrical units of brain circuitry that can be regulated or remodelled independently^1^. Synaptic input to a neuronal circuit is determined by dendritic spine density. In turn, the morphology of individual synapses is thought to reflect their function^1^,^2^. Abnormal spine density and morphology can underlie neurological disorders such as intellectual disabilities and autism, underscoring the importance of spine morphogenesis in brain functions.

Dendritic spines are highly dynamic, and their formation, turnover and morphology depend on synaptic activity from afferent inputs^3^. Acute activity-dependent spine growth and pruning appears to underlie the learning-related forms of synaptic plasticity, such as long-term potentiation (LTP) and long-term depression^4^,^5^. Neuronal activity is also required for dendritic spine maturation and maintenance during brain development. For example, exposing young rodents to an enriched environment increases dendritic spine density, whereas sensory deprivation increases spine turnover and reduces spine density in pyramidal neurons of the somatosensory cortex^6^,^7^. One mechanism underlying spine stabilization involves activation of the AMPA (α-amino-3-hydroxy-5-methyl-4-isoxazole propionic acid) class of excitatory ionotropic glutamate receptors by spontaneously released glutamate^8^,^9^. Blocking vesicular release by botulinum toxin reduces spine density in hippocampal tissue sections, which can be rescued by co-treatment with AMPA^8^. The pharmacological blockade of AMPA receptors by NBQX also reduces spine density in dissociated hippocampal neurons^10^. Conversely, AMPA receptor activation stabilizes dendritic spines by decreasing spine motility, which involves Ca^2+^ influx through voltage-activated Ca^2+^ channels^9^. However, the Ca^2+^-binding proteins involved in AMPA receptor-mediated spine maintenance remain unknown.

Dendritic spine formation and maintenance are mediated by the Rho family of small GTPases^11^. Rac1 and RhoA are the best-studied members that regulate spine dynamics; their activation facilitates and prevents dendritic spine formation, respectively^12^,^13^. Nevertheless, how synaptic activity differentially regulates Rac1 and RhoA to induce cytoskeletal rearrangement is largely unresolved. Different Ca^2+^-dependent signalling proteins may functionally link ionotropic glutamate receptors to specific Rho-GTPases. For example, Ca^2+^/calmodulin-dependent protein kinases (CaMKs) are an important class of Ca^2+^ effectors that trigger synaptic structural modifications^5^,^14^. CaMKs functionally link NMDA (*N*-methyl-D-aspartate) receptors to Rac1 activation via the phosphorylation of specific guanine nucleotide exchange factors (GEFs) such as Kalirin-7, TIAM1 and β-PIX^15^,^16^,^17^. In contrast, much less is known about how RhoA is regulated by excitatory glutamate receptors. The Rho-GEF Lfc (also known as GEF-H1) is one notable link between RhoA activity and glutamate receptors^18^. Lfc is inhibited by AMPA receptors; Lfc and RhoA inhibition is implicated in spine maintenance^10^. RhoA hyperactivity can lead to spine loss and impaired synaptic function, which may underlie the behavioural deficits in intellectual disabilities associated with loss-of-function oligophrenin-1 (OPHN1) mutations^19^,^20^,^21^. It is critical to explicate the Ca^2+^-dependent signalling protein(s) that regulate Lfc and RhoA activity downstream of glutamate receptors to better understand activity-dependent spine maintenance.

Besides CaMKs, the serine/threonine kinase cyclin-dependent kinase 5 (Cdk5) is crucial in the regulation of spine morphogenesis^22^,^23^,^24^. To understand how CaMK and Cdk5 signalling interact to regulate synaptic function, we used mass spectrometry to generate a library of synaptic Cdk5 substrates (unpublished observations). This exploratory study identified the protein CaMKv as a potential Cdk5 substrate.

In the present study, we show that CaMKv synthesis is induced by neuronal activity and sensory experience, and CaMKv is required for activity-dependent dendritic spine maintenance. In addition, CaMKv regulates spine morphogenesis in a Ca^2+^/calmodulin-dependent manner, and its role in spine maintenance is suppressed by Cdk5-mediated phosphorylation. CaMKv interacts with Lfc and increases spine number via RhoA inhibition. Furthermore, CaMKv knockdown in the hippocampal CA1 neurons of mice enhances locomotor activity and impairs spatial memory performance. These results indicate that CaMKv serves as convergence point for the transduction of Ca^2+^ signals to the neuronal cytoskeleton via RhoA; hence, CaMKv interacts with Cdk5 to regulate spine dynamics and contributes to normal synaptic transmission underlying brain function.

## Results

### Synaptic CaMKv synthesis is induced by neuronal activity

**Figure 1:**
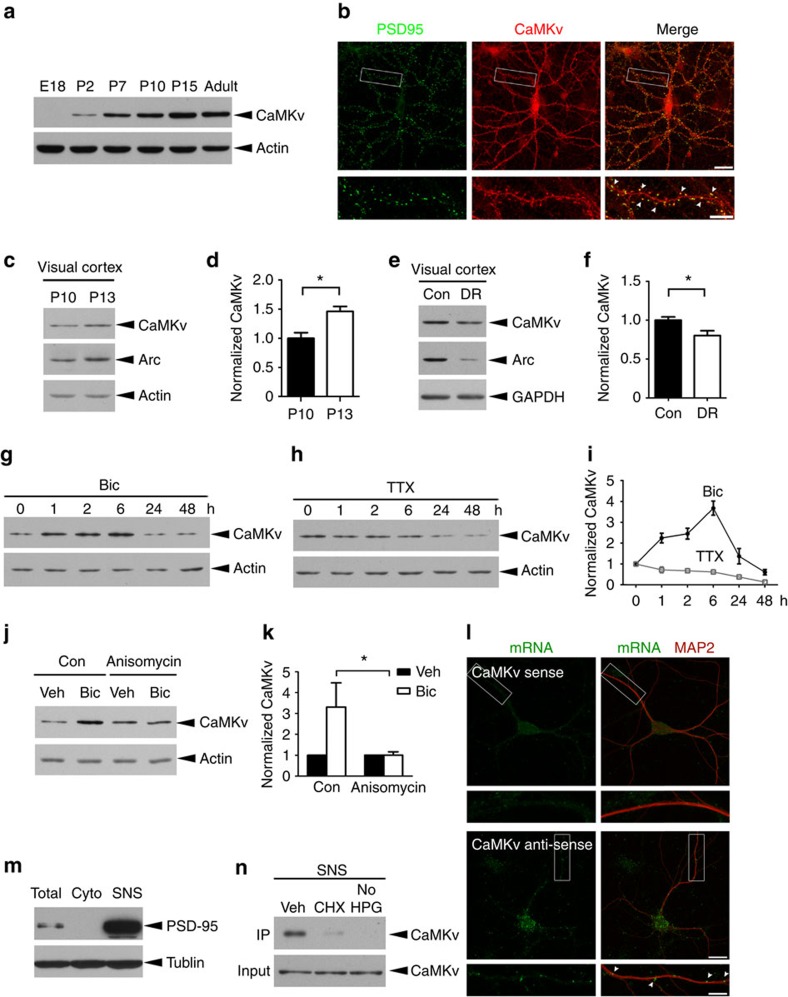
CaMKv protein synthesis at synapses is regulated by neuronal activity. (**a**) Western blot analysis of CaMKv expression in the mouse forebrain at the indicated stages. (**b**) Co-localization between CaMKv (red) and PSD-95 (green) puncta (arrowheads) in the dendrites of cultured hippocampal neurons (23 days *in vitro* (DIV)). Scale bars, 20 μm (upper); 10 μm (lower panels). (**c**) CaMKv expression in the mouse visual cortex before (postnatal day (P) 10) and after eye-opening (P13). Arc protein increase upon eye-opening served as a positive control. (**d**) Quantification of CaMKv protein normalized to that of actin (*n*=3 mice per condition, *P<0.05, Student's *t*-test). (**e**) CaMKv expression in the mouse visual cortex was reduced after dark rearing (DR). (**f**) CaMKv protein was quantified after normalization to that of GAPDH (*n*=11 mice per condition, *P<0.05, Student's *t*-test). (**g**,**h**) Cortical neurons (14–16 DIV) were treated with Bic (40 μM) or TTX (2 μM) for the indicated durations. (**i**) CaMKv expression (normalized to actin) was increased by Bic and decreased by TTX (*n*=3 independent experiments). (**j**,**k**) Bic-induced CaMKv expression required protein synthesis. Cortical neurons (14 DIV) were pretreated with anisomycin (40 μM) for 1 h and incubated with Bic (40 μM) or vehicle (Veh) for 1 h (*P<0.05, one-way analysis of variance, Bonferroni's multiple comparison test; *n*=3 independent experiments). (**l**) *camkv* mRNA was localized at the distal dendrites (>100 μm from cell bodies) of dissociated hippocampal neurons (23 DIV) as indicated by fluorescence *in situ* hybridization. Arrowheads indicate *camkv* mRNA (green) along MAP2-positive dendrites (red). Scale bars, 20 μm (upper); 10 μm (lower). (**m**) The postsynaptic marker PSD-95 was highly enriched in the synaptoneurosome fraction (SNS) but absent in the cytosolic fraction (Cyto). (**n**) CaMKv was locally translated in the synaptoneurosome as revealed by metabolic labelling; its synthesis was abolished by cycloheximide (CHX, 10 ng μl^−1^).

To identify novel Cdk5 substrates at neuronal synapses, synaptosome fractions were prepared from Cdk5 conditional knockout^25^ and wild-type (WT) control mice. Samples were analysed by mass spectrometry. Peptides containing phospho-serine or phospho-threonine specifically in the synaptosome were detected from the WT but not the Cdk5 conditional knockout mice. A putative Cdk5 substrate identified in this proteomic screen was CaMKv, a kinase-like calmodulin-binding protein of unknown function that is abundant in the nervous system^26^. Although the level of CaMKv messenger RNA (mRNA) remains relatively constant, CaMKv protein expression was upregulated throughout brain development and neuronal maturation (Fig. 1a; Supplementary Fig. 1a). Consistent with CaMKv detection in the synaptosome in our proteomic screen, CaMKv was present in the synaptic plasma membrane and postsynaptic density (PSD) of the adult rat brain (Supplementary Fig. 1b). Immunofluorescence staining further demonstrated the co-localization of CaMKv puncta with the postsynaptic scaffold protein PSD-95 (Fig. 1b).

A recent proteomic study revealed the significant reduction of CaMKv proteins in sensory-deprived barrel cortex *in vivo*^27^, suggesting that CaMKv may play a role in activity-dependent synapse development. Toward this end, we first determined whether sensory experience drives CaMKv expression in the visual cortex during development. As expected, CaMKv protein in the visual cortex was elevated upon eye-opening and suppressed by dark rearing (DR; Fig. 1c–f). These findings indicate that CaMKv expression depends on sensory experience *in vivo*. To verify the regulation of CaMKv expression by synaptic activity, we enhanced or blocked neuronal activity in primary cortical neurons by treatment with bicuculline (Bic) and tetrodotoxin (TTX), respectively. Indeed, CaMKv expression was bi-directionally regulated on Bic and TTX treatment (Fig. 1g–i). Moreover, the induction of CaMKv synthesis was abolished by anisomycin (a protein synthesis inhibitor) but not actinomycin D (a transcription inhibitor; Fig. 1j,k; Supplementary Fig. 1c), and the level of *camkv* mRNA was not changed by Bic treatment (Supplementary Fig. 1d); this indicates that the increase in CaMKv is independent of transcription but dependent on the translation of preexisting mRNA.

Increasing evidence suggests that protein synthesis in dendrites is an important mechanism in neural circuitry development and plasticity. Indeed, *camkv* mRNA is among the ∼2,500 transcripts detected in the hippocampal neuropil^28^. To verify the dendritic localization of *camkv* mRNA, fluorescence *in situ* hybridization was performed on dissociated hippocampal neurons. The *camkv* transcripts appeared as distinct puncta, that is, RNA granules, which were detected in distal dendrites (Fig. 1l). To confirm the local synthesis of synaptic CaMKv, synaptoneurosomes were prepared for metabolic labelling, in which non-canonical amino acids incorporated into newly synthesized proteins were biotin-conjugated after a click reaction^29^. Subsequent immunoprecipitation by Streptavidin beads pulled down CaMKv, indicating that it was locally synthesized at the synapse (Fig. 1m,n). Collectively, these findings suggest that CaMKv is a synaptic protein whose synthesis can be driven by local synaptic activity.

### CaMKv regulates dendritic spine density through Ca^2+^/calmodulin

**Figure 2:**
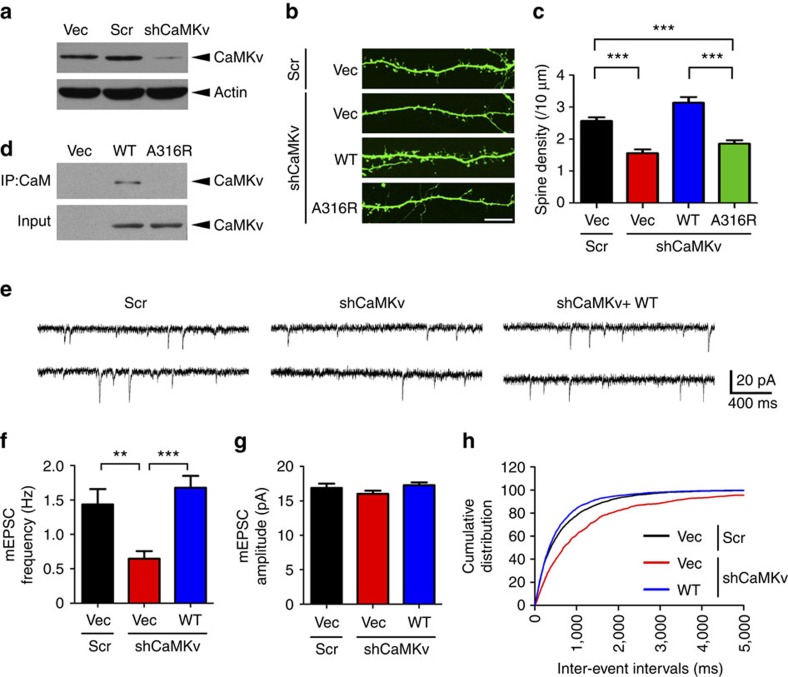
CaMKv is required for dendritic spine maintenance and synaptic transmission. (**a**) Western blot analysis of CaMKv expression in cortical neurons transfected with pSUPER vector (Vec), shRNAs (shCaMKv) or the corresponding scrambled control (Scr). (**b**,**c**) CaMKv knockdown significantly decreased dendritic spine density. The spine loss was rescued by co-expressing the RNAi-resistant wild-type (WT) CaMKv but not the A316R mutant (scale bar, 10 μm; *n*=39 dendrites per condition, ***P<0.001, one-way analysis of variance (ANOVA), Tukey's multiple comparison test). (**d**) The pull-down experiment indicated that WT CaMKv but not the A316R mutant interacted with calmodulin (CaM). (**e**–**h**) CaMKv was required for synaptic transmission. (**e**) Representative miniature excitatory postsynaptic current (mEPSC) traces. (**f**,**h**) CaMKv knockdown significantly decreased mEPSC frequency, which was restored by co-expressing WT CaMKv. (**g**) No significant difference in mEPSC amplitude was observed among conditions (Scr: *n*=27 neurons, shCaMKv: *n*=29 neurons, WT CaMKv rescue: *n*=28 neurons, **P<0.01, ***P<0.001, one-way ANOVA, Tukey's multiple comparison test).

The subcellular localization of CaMKv mRNA and protein as well as the tight regulation of CaMKv expression by synaptic activity suggest that it may play a key role at synapses. As CaMKs are implicated in spine morphogenesis^17^, we determined whether the depletion of CaMKv affects dendritic spine density and morphology. Two short hairpin RNAs (shRNAs) designed for CaMKv knockdown in cultured neurons were effective (Fig. 2a; Supplementary Fig. 2a). Dissociated hippocampal neurons (14 days *in vitro* (DIV)) were transfected with green fluorescent protein (GFP) construct together with either CaMKv shRNA or the corresponding scrambled shRNA control. CaMKv knockdown by either shRNA significantly reduced dendritic spine density (Fig. 2b,c; Supplementary Fig. 2b,c). Co-transfection of RNA interference (RNAi)-resistant CaMKv constructs with the shRNA in cultured neurons rescued the reduced spine density, suggesting that the observed spine defects were due to CaMKv depletion (Fig. 2b,c). To ascertain whether CaMKv plays a permissive or instructive role in spine morphogenesis, we examined the effect of CaMKv overexpression. Compared with the vector control, CaMKv overexpression in dissociated hippocampal neurons significantly increased dendritic spine number, suggesting that CaMKv regulates spine morphogenesis in a gain-of-function manner (Supplementary Fig. 2d,e).

Amino-acid alignment indicated that CaMKv contains a conserved calmodulin-binding site at Ala-316, cognate to Ala-302 of CaMKII, which is required for calmodulin binding^30^. To determine whether CaMKv function depends on Ca^2+^/calmodulin binding, we generated a CaMKv A316R mutant, which failed to bind calmodulin in the pull-down assay (Fig. 2d). Notably, the reduced spine density upon CaMKv knockdown could only be rescued by WT CaMKv and not the A316R mutant (Fig. 2b,c). Moreover, unlike WT CaMKv, A316R mutant overexpression did not increase spine density (Supplementary Fig. 2d,e). Sequence alignment suggests that CaMKv is a pseudokinase; accordingly, CaMKv does not exhibit kinase activity *in vitro*^26^,^31^. To corroborate the *in vitro* findings, we determined whether CaMKv functions in a kinase-independent manner by generating a mutation in the ATP-binding domain of CaMKv (K48R). The rescue experiment showed that the CaMKv-K48R mutant reversed the spine defects caused by the CaMKv shRNA (Supplementary Fig. 2f,g). These findings collectively indicate that the promotion of spine formation/maintenance by CaMKv is independent of kinase activity but requires Ca^2+^/calmodulin binding.

Changes in spine density are closely correlated with synaptic transmission efficacy. To determine whether CaMKv knockdown regulates functional connectivity between neurons, the frequency and amplitude of miniature excitatory postsynaptic currents (mEPSCs) were measured in dissociated hippocampal neurons on CaMKv knockdown. Consistent with spine density reduction, CaMKv knockdown reduced mEPSC frequency without affecting amplitude. Importantly, the mEPSC frequency reduction was completely rescued by co-transfection of RNAi-resistant CaMKv (Fig. 2e–h), indicating that CaMKv is essential for spontaneous excitatory neurotransmission.

### CaMKv regulates dendritic spine density through RhoA inhibition

**Figure 3:**
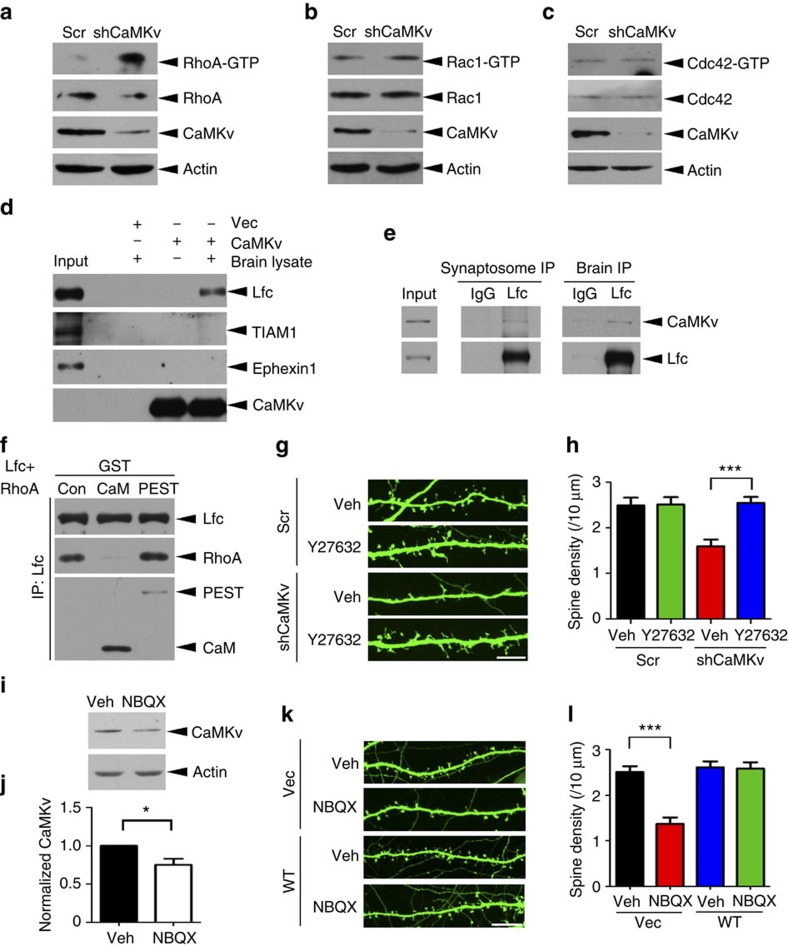
CaMKv regulates spine morphogenesis via RhoA inhibition. (**a**–**c**) CaMKv knockdown dramatically increased RhoA activity but not Rac1 or Cdc42. (**d**) The RhoA-GEF Lfc, but not the other GEFs TIAM1 or Ephexin1, was pulled down from the synaptosome by FLAG-tagged CaMKv. (**e**) CaMKv was co-immunoprecipitated with Lfc in brain homogenate and synaptosome fractions. Immunoprecipitation by IgG served as the negative control. (**f**) FLAG-tagged Lfc and HA-tagged RhoA were co-expressed in HEK293T cells, and the lysate was subsequently incubated with recombinant GST-tagged CaMKv CaM-binding domain (CaM) or the CaMKv PEST domain. Lfc–RhoA interaction was specifically disrupted by the CaMKv CaM-binding domain but not the PEST domain. (**g**,**h**) Hippocampal neurons transfected with CaMKv shRNA or Scr were treated with the ROCK inhibitor Y27632 (10 μM) or vehicle (Veh) at 17 DIV for 6 h. Y27632 abolished the spine loss induced by CaMKv shRNA (scale bar, 10 μm; *n*=30 dendrites per condition, ***P<0.001, one-way analysis of variance (ANOVA), Tukey's multiple comparison test). (**i**,**j**) Cultured hippocampal neurons were treated with the AMPA receptor blocker NBQX (20 μM) from 9 to 16 DIV. NBQX reduced CaMKv expression (*n*=3 independent experiments, *P<0.05, Student's *t*-test). (**k**,**l**) Hippocampal neurons transfected at 7 DIV with the indicated constructs were treated with NBQX (20 μM) or vehicle (Veh) from 9 to 16 DIV. The spine loss on long-term AMPA receptor blockade was rescued by CaMKv overexpression (scale bar, 10 μm; *n*=30 dendrites per condition, ***P<0.001, one-way ANOVA, Tukey's multiple comparison test).

Next, we investigated the mechanism by which CaMKv regulates spine morphogenesis. As the Rho family of small GTPases has been implicated to regulate spine density^10^,^12^,^13^, we examined RhoA, Rac1 and Cdc42 activity after transfection of cultured cortical neurons with scrambled or CaMKv shRNA (Fig. 3a–c). Among the three small GTPases, RhoA activity was most drastically induced after CaMKv depletion (Fig. 3a; Supplementary Fig. 3a). Consistent with the change in RhoA activity and loss of dendritic spines, CaMKv knockdown also reduced the proportion of filamentous actin (Supplementary Fig. 3b,c).

Next, we investigated how CaMKv suppresses RhoA activity. The activity of the Rho family of small GTPases requires binding to specific GEFs. Accordingly, the RhoA-specific GEF Lfc was pulled down by CaMKv (Fig. 3d). This binding was specific; CaMKv was not associated with the other Rho-GEF Ephexin1 or Rac1-GEF TIAM1 (Fig. 3d). Endogenous Lfc also interacts with CaMKv in the brain homogenate and synaptosome fractions (Fig. 3e). As CaMKv knockdown resulted in RhoA activity induction, CaMKv may inhibit RhoA through competitive binding to Lfc. To test this, co-immunoprecipitation between RhoA and Lfc was performed in the presence of GST-fusion proteins encoding different CaMKv domains. The presence of the CaMKv calmodulin-binding domain inhibited the interaction between Lfc and RhoA, while the PEST domain had no effect (Fig. 3f). These observations suggest that CaMKv blocks RhoA activity by preventing its interaction with Lfc. To further evaluate the function of CaMKv-RhoA signalling in dendritic spine maintenance, the effect of the Y27632 inhibitor of the Rho-associated protein kinase ROCK on CaMKv shRNA-dependent changes in spine density was assessed. While treatment with Y27632 alone did not affect spine density, Y27632 abolished the effect of CaMKv knockdown on spine density (Fig. 3g,h). These results demonstrate that CaMKv regulates spine maintenance through RhoA inhibition.

The suppression of the Lfc/RhoA pathway by AMPA receptors is implicated in dendritic spine maintenance^10^. Given that CaMKv expression is upregulated by synaptic activity, CaMKv may be a key signalling protein that transduces the signals from AMPA receptors to suppress RhoA during activity-dependent spine maintenance. Corroborating this, the AMPA receptor blocker NBQX attenuated CaMKv expression in neurons (Fig. 3i,j). Importantly, CaMKv overexpression completely rescued NBQX-induced spine loss (Fig. 3k,l). These findings collectively reveal that activity-dependent CaMKv expression and the subsequent inhibition of Lfc/RhoA are required to mediate AMPA receptor function in spine maintenance.

### Cdk5-mediated phosphorylation inhibits CaMKv function in spine maintenance

**Figure 4:**
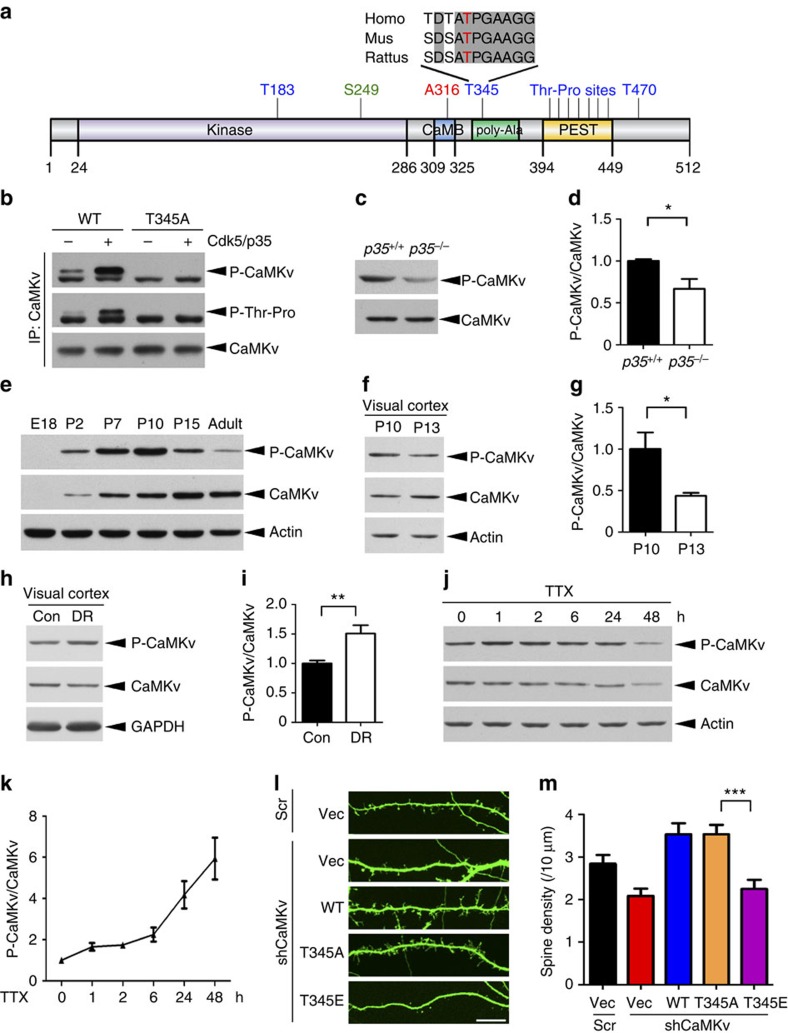
CaMKv phosphorylation at Thr345 by Cdk5 and its role in spine morphogenesis. (**a**) Schematic diagram showing the different phospho-serine and phospho-threonine residues of CaMKv. Thr345 is conserved in the human, mouse, and rat orthologues. (**b**) Phospho-specific antibody against CaMKv Thr345 (P-CaMKv) revealed the induction of Thr345 phosphorylation of FLAG-tagged CaMKv by recombinant Cdk5/p35. The signal was abolished in the T345A phospho-deficient CaMKv mutant. (**c**,**d**) Thr345 phosphorylation was significantly reduced in *p35*^−/−^ adult mouse brains. (*p35*^+/+^: *n*=4 mice, *p35*^−/−^: *n*=5 mice, *P<0.05, Student's *t*-test). (**e**) CaMKv phosphorylation and expression levels in the mouse forebrain at different developmental stages. (**f**,**g**) CaMKv phosphorylation decreased significantly in the visual cortex after eye-opening (P13). CaMKv phosphorylation levels were normalized to that of total CaMKv (*n*=3 mice per condition, *P<0.05, Student's *t*-test). (**h**,**i**) CaMKv phosphorylation significantly increased in the mouse visual cortex after DR (*n*=11 mice per condition, **P<0.01, Student's *t*-test). (**j**,**k**) CaMKv phosphorylation increased on activity blockade. Cortical neurons (14–16 DIV) were treated with TTX (2 μM) for the indicated durations and quantified after normalization to total CaMKv (*n*=3 independent experiments). (**l**,**m**) The phospho-mimetic CaMKv-T345E mutant failed to rescue the spine defects on CaMKv knockdown by shRNA (scale bar, 10 μm; *n*=33 dendrites per condition, ***P<0.001, one-way analysis of variance, Tukey's multiple comparison test).

We initially identified CaMKv as a putative substrate of Cdk5 from a proteomic screen. CaMKv phosphorylation by Cdk5 was confirmed by *in vitro* phosphorylation assay; the presence of recombinant Cdk5 and its activator p35 significantly increased CaMKv ^32^P-ATP incorporation (Supplementary Fig. 4a). Cdk5/p35 co-expression specifically induced the Thr, but not Ser, phosphorylation of CaMKv (Supplementary Fig. 4b). In addition, CaMKv was co-immunoprecipitated with Cdk5 and p35 from mouse brain lysates, indicating their interaction *in vivo* (Supplementary Fig. 4c). To verify CaMKv phosphorylation by Cdk5 *in vivo*, we mapped the major phosphorylation sites on CaMKv; there are 10 Thr–Pro sites, and 7 are localized within the PEST domain (Fig. 4a). The Thr residues were individually or collectively mutated to Ala via site-directed mutagenesis. Thr345 located within the poly-Ala region was identified as the major site of CaMKv phosphorylation by Cdk5 (Supplementary Fig. 4d). More importantly, using an antibody that specifically recognizes Thr345 (Fig. 4b), we observed significant reduction of phospho-Thr345 CaMKv in p35 knockout mouse brains (Fig. 4c,d). These findings collectively indicate that Cdk5 phosphorylates CaMKv at Thr345 in the brain.

While CaMKv expression increased in the mouse brain during development, Thr345 phosphorylation drastically decreased from postnatal day 10, which was consistent with the change of Cdk5 activity along development (Fig. 4e; Supplementary Fig. 4e). CaMKv phosphorylation in the mouse visual cortex was reduced after eye-opening, but was elevated on DR (Fig. 4f–i). Moreover, TTX increased Thr345 phosphorylation in cortical neurons, suggesting that the elevated phosphorylation was due to neuronal activity blockade (Fig. 4j,k). Given that CaMKv expression was upregulated by synaptic activity (Fig. 1c–k), the reciprocal regulation suggests that the phosphorylation may inhibit CaMKv function. To confirm this, we generated RNAi-resistant phospho-deficient T345A and phospho-mimetic T345E CaMKv mutants, and co-transfected them with CaMKv shRNA in a rescue experiment (Supplementary Fig. 4h–j). Introducing either the WT or phospho-deficient T345A CaMKv mutant rescued the loss of dendritic spines caused by CaMKv shRNA in hippocampal neurons. However, co-expression of the phospho-mimetic T345E failed to restore the defects in spine density after CaMKv depletion (Fig. 4l,m), suggesting that CaMKv phosphorylation by Cdk5 at Thr345 inhibits its function.

### CaMKv is required for synaptic transmission and late-phase LTP

**Figure 5:**
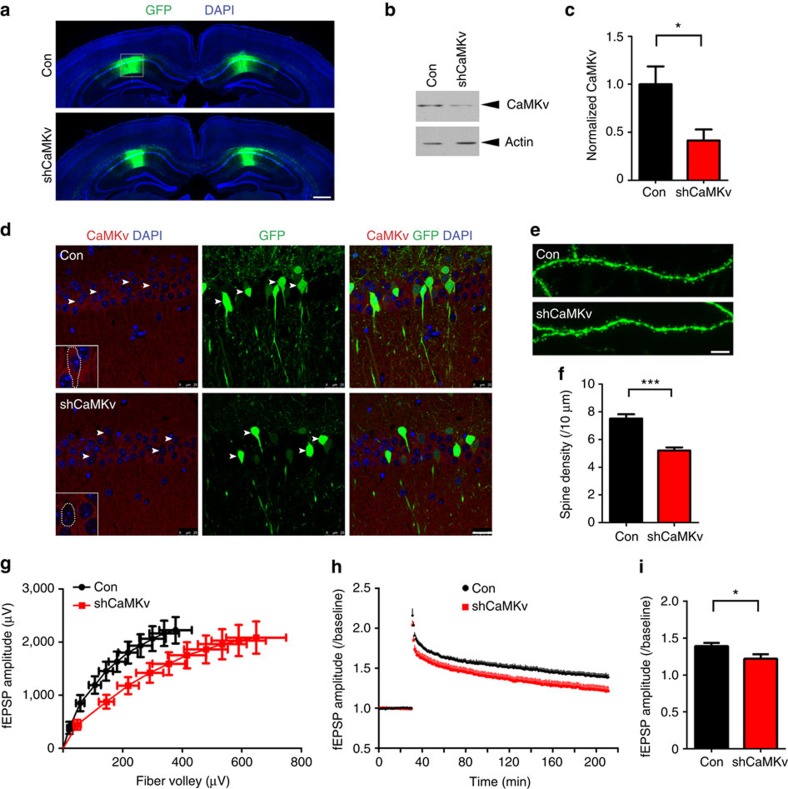
CaMKv knockdown *in vivo* reduces spine density and impairs basal synaptic neurotransmission and L-LTP. (**a**) Representative immunostained coronal sections from mice subjected to bilateral lentivirus injections (scale bar, 500 μm). (**b**,**c**) The indicated GFP-positive CA1 region was dissected to examine CaMKv levels. CaMKv knockdown (shCaMKv) in the hippocampal CA1 region significantly reduced CaMKv expression (Con: *n*=4 mice, shCaMKv: *n*=6 mice, *P<0.05, Student's *t*-test). (**d**) Hippocampal CA1 pyramidal neurons were transduced by control or lentiviral vectors expressing CaMKv shRNA. CaMKv knockdown *in vivo* reduced CaMKv expression (red) in CA1 neurons (scale bar, 25 μm; arrowheads: GFP-positive neurons; dotted area: magnified images of infected neurons). (**e**,**f**) CaMKv knockdown *in vivo* significantly reduced dendritic spine density. (scale bar, 20 μm; Con: *n*=34 dendrites, shCaMKv: *n*=21 dendrites, ***P<0.001, Student's *t*-test; green; GFP, red: CaMKv, blue: 4,6-diamidino-2-phenylindole (DAPI)). (**g**) CaMKv knockdown in hippocampal CA1 neurons inhibited basal synaptic neurotransmission. The input–output curve was altered in the infected CA1 region after CaMKv knockdown (Con: *n*=8 mice, shCaMKv: *n*=9 mice). (**h**) CaMKv knockdown impaired the L-LTP. (**i**) The field excitatory postsynaptic potential (fEPSP) amplitude (normalized to baseline) was significantly reduced in hippocampi injected with CaMKv shRNA (Con: *n*=8 mice, shCaMKv: *n*=9 mice, *P<0.05, Student's *t*-test).

Given that CaMKv depletion resulted in dendritic spine loss and impaired synaptic transmission in cultured hippocampal neurons, we examined the consequences of CaMKv knockdown in the hippocampus *in vivo*. Lentivirus containing CaMKv shRNA or control virus was stereotaxically injected into the hippocampal CA1 region. The GFP signal was largely confined to the injected CA1 region, suggesting that CaMKv knockdown was spatially restricted to the injected area; the knockdown efficiency was confirmed by the significant reduction of CaMKv protein expression (Fig. 5a–c). Immunohistochemistry further confirmed the reduced CaMKv immunoreactivity in GFP-positive neurons in the shRNA-injected mouse sections (Fig. 5d).

We subsequently determined whether CaMKv depletion affects the spine morphogenesis of hippocampal neurons *in vivo*. Consistent with the findings in dissociated hippocampal neurons, CaMKv shRNA virus injection into the mouse hippocampus significantly decreased spine density in CA1 neurons compared with the control virus injection (Fig. 5e,f). Furthermore, delivery of ROCK inhibitor *in vivo* restored the spine density after CaMKv knockdown (Supplementary Fig. 5a,b). These findings confirm that CaMKv is required to maintain dendritic spine density through the inhibition of RhoA *in vivo*. Extracellular field recording of CA1 neurons also revealed attenuated synaptic transmission indicated by the ratio of the field excitatory postsynaptic potential (fEPSP) amplitude to fibre volley (Fig. 5g). Therefore, CaMKv function in spine maintenance contributes to normal synaptic transmission in mouse hippocampus.

Long-lasting synaptic plasticity such as late-phase LTP (L-LTP) requires *de novo* protein synthesis, which is the molecular basis of memory storage in the brain. As CaMKv is locally translated at synapses (Fig. 1m,n), we investigated whether CaMKv contributes to L-LTP. L-LTP was significantly attenuated after CaMKv knockdown, while NMDA receptor-dependent long-term depression was unchanged (Fig. 5h,i; Supplementary Fig. 5c). These findings suggest that CaMKv is required for both synaptic transmission and protein synthesis-dependent LTP in the hippocampus.

### Hippocampal CaMKv knockdown results in locomotor and memory deficits

**Figure 6:**
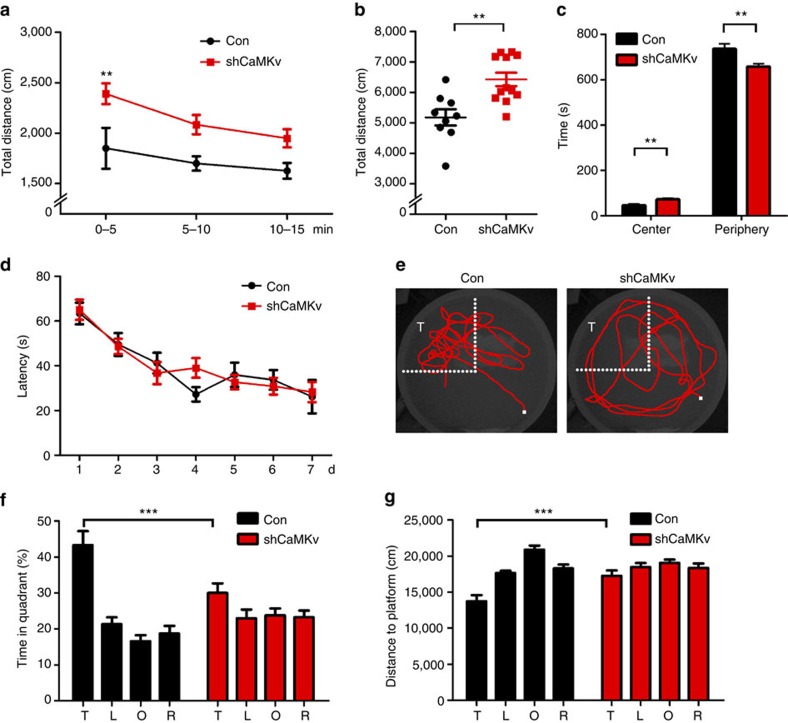
CaMKv knockdown in hippocampal CA1 neurons leads to hyperactivity and reduced MWM performance. (**a**–**c**) CaMKv knockdown mice exhibited hyperlocomotion in the open-field test, significantly increased total running distance and central/total duration (Con: *n*=9 mice, shCaMKv: *n*=12 mice, **P<0.01, Student's *t*-test). (**d**–**g**) CaMKv knockdown reduced MWM performance. (**d**) Mice were trained in the MWM and tested in a probe trial on day 8. The acquisition over 7 days (four trials per day) was similar between groups. (**e**) Representative swimming paths of a control mouse and a CaMKv knockdown mouse are shown. (**f**) In the probe trial, control mice spent significantly more time in the target quadrant (T) than any other quadrant (T versus L, T versus O or T versus R, ***P<0.001, one-way analysis of variance (ANOVA), Tukey's multiple comparison test). The CaMKv knockdown mice spent comparable time in T as the other quadrants at the level of chance (T versus L, T versus O or T versus R, *P*>0.05, one-way ANOVA, Tukey's multiple comparison test). Time spent in T was significantly lower in the CaMKv knockdown mice than the controls (Con: *n*=14 mice, shCaMKv: *n*=18 mice, ***P<0.001, two-way ANOVA, genotype × platform: *F*_(3,90)_=5.49, *P*<0.01, genotype: *F*_(1,30)_=−0.4835, platform: *F*_(3,90)_=16.05, *P*<0.0001, Bonferroni's multiple comparison test). (**g**) The cumulative distance of the swimming path to the platform was greater in CaMKv knockdown mice than the controls (Con: *n*=14 mice, shCaMKv: *n*=18 mice, ***P<0.001, two-way ANOVA, genotype × platform: *F*_(3,120)_=6.48, *P*<0.001, genotype: *F*_(1,120)_=−2.16, platform: *F*_(3,120)_=17.92, *P*<0.0001, Bonferroni's multiple comparison test).

Given that CaMKv is important for the structure and function of hippocampal synapses, we investigated whether hippocampal-specific CaMKv knockdown affects behavioural performance. CaMKv was specifically knocked down in the CA1 neurons of the dorsal hippocampus in mice using viral-mediated CaMKv shRNA. The impacts on home-cage activity, open-field exploration and the Morris water maze (MWM) performance were assessed. Mice injected with control virus served as controls. In the open-field test, CaMKv knockdown mice were hyperactive, as indicated by increased total moving distance (Fig. 6a,b). Consistently, home-cage activity was also increased in CaMKv knockdown mice during the initial phase (Supplementary Fig. 5e,f). CaMKv knockdown mice also spent significantly more time in the central area of the open field (Fig. 6c).

The effect of CaMKv knockdown in hippocampal CA1 neurons on spatial memory formation was investigated using the MWM paradigm (Fig. 6d–g). Acquisition was comparable between the CaMKv shRNA-injected mice and the controls (Fig. 6d). However, in the MWM probe trial, control mice exhibited spatial learning and memory capability, spending significantly more time in the target quadrant than any other quadrant (Fig. 6f). In contrast, the CaMKv knockdown mice exhibited no significant spatial learning or memory capability, spending comparable time in the target quadrant as in the other quadrants (that is, at the level of chance; Fig. 6f). Accordingly, the cumulative distance from any given point of the swimming path to the platform was greater in CaMKv knockdown mice than in controls (Fig. 6g).

Emerging evidence suggests that hippocampus contributes to spatial encoding in working memory through synaptic changes^32^,^33^. We therefore also determined whether CaMKv is involved in the processing of working memory. Indeed, CaMKv knockdown mice exhibited decreased alternation rate in a T-maze spontaneous alternation task (Supplementary Fig. 5d), indicating that CaMKv in the hippocampus is required for the encoding of information into working memory. These results collectively show that CaMKv knockdown in the CA1 neurons of the dorsal hippocampus induces spatial memory deficits, underlining an important role of CaMKv in learning and memory.

## Discussion

The maintenance and pruning of synaptic connectivity are tightly regulated by neuronal activity. Although AMPA receptor activation promotes dendritic spine maintenance, the underlying mechanism is unclear. The present study identified the Ca^2+^/calmodulin-binding protein CaMKv as a synaptic protein whose expression is regulated by AMPA receptors. Importantly, despite being classified as a pseudokinase, CaMKv plays important roles in regulating dendritic spine morphogenesis as well as synaptic transmission and plasticity, which are crucial for learning and memory processes. Numerous pseudoenzymes including pseudokinases are encoded in the genome^31^, but few have been characterized or further demonstrated to contribute to physiological functions. Therefore, the present study demonstrates an unusual signalling pathway mediated by a pseudokinase in the regulation of synaptic function.

CaMKv was originally identified as a vesicle-associated kinase-like calmodulin-binding protein abundantly expressed in the central nervous system^26^. Despite sharing homology with CaMKII-α and CaMKII-β, CaMKv lacks a few amino-acid residues universally conserved among different members of the CaMK family and does not possess kinase activity^26^. Hence, CaMKv may be the only pseudokinase of the CaMK family^31^. While the function of CaMKv is unknown, a large-scale transcriptomic study revealed *camkv* as one of the many mRNA transcripts present in the synaptic neuropil^28^. In addition, a quantitative proteomics study shows that CaMKv is downregulated at the synapse on sensory deprivation^27^. Consistent with the emerging importance of the interplay between local dendritic translation and the regulation of actin cytoskeleton dynamics in spine morphogenesis^3^,^34^,^35^, our findings suggest that CaMKv acts as a molecular link between AMPA receptor signalling to RhoA-dependent actin dynamics and organization (Supplementary Fig. 6). Hence, we hypothesize that AMPA receptor activation promotes dendritic CaMKv synthesis likely through elevated brain-derived neurotrophic factor release as reported previously^34^. Supporting this hypothesis, CaMKv expression was increased in hippocampal neurons after brain-derived neurotrophic factor treatment (Supplementary Fig. 1e,f). The continued CaMKv synthesis on AMPA receptor activation controls RhoA activity, at least in part through the inhibition of the Lfc–RhoA interaction; in turn, the balance of RhoA activity is essential for dendritic spine stability.

While dendritic spine maintenance is crucial for neural network stability, spine pruning is ongoing in both the developing and adult brain, which underlies the remodelling of neuronal connectivity through experience. If CaMKv promotes spine maintenance, termination of its action may be required during spine pruning. The present study demonstrates that CaMKv phosphorylation by Cdk5 is a major regulatory mechanism that inhibits CaMKv function. Consistent with this hypothesis, treating neurons with ephrin-A1, which activates the receptor EphA4 and induces spine retraction via Cdk5 and RhoA activation^22^, also increased CaMKv phosphorylation at Thr345 (Supplementary Fig. 4f,g). This suggests that ephrin-A induces the Cdk5 phosphorylation of CaMKv during spine pruning, consequently contributing to dendritic spine loss.

The tight regulation of RhoA activity is crucial for proper brain function, as exemplified by its hyperactivation caused by loss-of-function mutations of the RhoA-GAP OPHN1. Since *ophn1* was the first intellectual disability-associated gene to be linked to the Rho-GTPase signalling pathway^19^, multiple loss-of-function mutations of the gene have been identified^36^. OPHN1 knockdown or knockout is associated with reduced spine density and excitatory synaptic transmission in hippocampal neurons in mice^20^,^21^. These synaptic defects are accompanied by behavioural deficits including novelty-driven hyperactivity in the open-field test and impaired spatial memory in the MWM^21^. These findings indicate that RhoA overactivation in hippocampal neurons is detrimental and leads to hyperactivity and impaired memory formation. After CaMKv is knocked down specifically in adult CA1 hippocampal neurons, the mice also exhibit hyperactivity and spatial memory defects in the MWM compared with the controls. Therefore, our findings are concordant with the idea that RhoA activity dysregulation and the subsequent impairment in hippocampal synaptic transmission lead to abnormal explorative and learning behaviours. It is noteworthy that the human *camkv* gene is located in chromosome 3 (3p21.31), and copy-number variants and microdeletion of this chromosome region have been reported in individuals with autism, intellectual disability and developmental delays^37^,^38^,^39^,^40^. This suggests a possible link between CaMKv dysregulation and neurodevelopmental disorders associated with intellectual disability, which is of immense interest and warrants further investigation.

## Methods

### Constructs and antibodies

Mouse CaMKv cDNA was subcloned into the pcDNA3 vector. The shRNAs for mouse CaMKv were designed online (http://sirna.wi.mit.edu/). The RNAi target sequences were shCaMKv (5′-gaactcaaagattgtcatc-3′) and shCaMKv-2 (5′-gccaagaacgagattggaa-3′), whereas the corresponding scrambled shRNAs were Scr (5′-gaaatatcgacccgttata-3′) and Scr-2 (5′-ggaaaggccgaacaattga-3′). The complementary oligonucleotides were annealed, subcloned into the pSUPER vector and expressed in cortical neurons by nucleofection to confirm the knockdown efficiencies.

CaMKv mutants were generated using the QuikChange mutagenesis kit (Agilent Technologies) according to the manufacturer's instructions. The primers for the CaMKv mutants were described as Supplementary Table 1.

Antibodies specific to CaMKv (1:100–2,000, S-17, D-18), Arc (1:500, C-7), Cdk5 (1:5,000, C-8, DC-17), p35 (1:2,000, C-19), CaM (1:500, FL-149), RhoA (1:500, 26C4), Cdc42 (1:500), TIAM1 (1:1,000) and GAPDH (1:10,000) were purchased from Santa Cruz Biotechnology; antibodies against β-actin (1:5,000), MAP2 (1:2,000) and FLAG (1:1,000–5,000, M2) were purchased from Sigma; antibodies against PSD-95 (1:5,000), Lfc (1:1,000, GEF-H1), phospho-Rb (1:1,000), proline-directed phospho-serine (1:1,000) and phospho-threonine (1:1,000) were purchased from Cell Signaling Technology; the antibody against GST (1:10,000) was from GE Healthcare; anti-Rac1 (1:500) was from BD Biosciences; and anti-GFP IgG2a (1:500) was from Invitrogen Life Technologies. The Lfc antibody (2 μg for each reaction, GTX125893) for co-immunoprecipitation was from Genetex. The antibody against phospho-WAVE1 (1:2,000, S441) was gift from P. Greengard (Rockefeller University). The antibody against phospho-TrkB (1:2,000) was prepared as described previously^23^. The rabbit polyclonal antibody against phospho-Thr345 CaMKv (1:500) was raised from the synthetic peptide (CTQSASDAApTPGAAGG) and purified by a Sulfolink immobilization kit for peptides (Thermo Fisher Scientific).

### Cell cultures and transfection

HEK293T cells (American Type Culture Collection) were cultured in DMEM with 10% heat-inactivated fetal bovine serum, 50 U ml^−1^ penicillin and 100 μg ml^−1^ streptomycin, and incubated at 37 °C in a humidified atmosphere with 5% CO_2_. HEK293T cells were transfected with Lipofectamine Plus transfection reagents according to the manufacturer's instructions. Cells were collected for further analysis at 24 h post transfection.

### Primary neuron culture

Sprague-Dawley rat embryos were killed at embryonic day 18. The cortical neurons were seeded on a culture plate or culture dishes (1 × 10^7^ cells per 100-mm dish, 4 × 10^6^ cells per 60-mm dish) coated with 100 μg ml^−1^ poly-L-lysine. Meanwhile, hippocampal neurons were seeded on 18-mm coverslips (1.5 × 10^5^ or 0.5 × 10^5^ cells per coverslip) coated with 1 mg ml^−1^ poly-D-lysine. The neurons were incubated at 37 °C in a humidified atmosphere with 5% carbon dioxide. Hippocampal neurons at 14 DIV were transfected with different plasmids plus EmGFP using Ca^2+^ phosphate precipitation^22^ and fixed at 17 DIV for morphological analysis.

### Protein immunoprecipitation and western blot analysis

The visual cortical tissues were collected at the indicated time points as described previously^41^. For the DR experiments, litters regardless of gender were divided into two groups after birth: the control animals were raised in a normal light/dark cycle from birth, and the DR animals were moved into a photon-free chamber at postnatal day (P) 1–2. Both groups were killed, and their visual cortices were collected 1 day after eye-opening of the control mice^42^.

For most biochemical studies, cortical neurons were lysed by radioimmunoprecipitation assay plus various protease and phosphatase inhibitors. For co-immunoprecipitation, HEK293T cells and cultured cortical neurons were lysed in buffer A (20 mM Tris (pH 8.5), 50 mM NaCl, 1 mM EDTA, 1 mM NaF and 0.5% Nonidet P-40 (v/v)) with protease and phosphatase inhibitors. Mouse brains were homogenized in PBS plus protease and phosphatase inhibitors. Synaptosome and PSD fractions were prepared as described previously^23^. Co-immunoprecipitation of HEK293T cells or whole-brain lysate was performed in buffer A. Lysate (1 mg for HEK293T cells, 2 mg for cultured cortical neurons or brain homogenate) was incubated with the corresponding antibody (1–2 μg) at 4 °C for either 2 h or overnight and subsequently incubated with 50 μl protein G-Sepharose at 4 °C for 1 h. For FLAG-tagged proteins, FLAG-conjugated beads (Sigma) were applied for the immunoprecipitation. Samples were washed with buffer A and resuspended in SDS sample buffer. Co-immunoprecipitated proteins were detected by western blotting. Densitometric quantification of protein band intensity was performed using Photoshop (Adobe). Full-length blots are shown in Supplementary Fig. 7.

### Mass spectrometry

Crude synaptosome proteins were extracted from the hippocampal CA1 region of Cdk5 conditional knockout mice^25^ and prepared for phosphorylation analysis by liquid chromatography–tandem mass spectrometry as described previously^43^. In brief, the proteins were precipitated by trichloroacetic acid (Sigma) and dissolved in 100 mM Tris–HCl (pH 8.5) containing 8 M urea (Sigma). The analysis was performed with a LTQ-Orbitrap mass spectrometer (Thermo Fisher Scientific). To accurately estimate peptide probabilities and false discovery rates, we used a decoy database containing the reversed sequences of all proteins appended to the target database^44^. Tandem mass spectra were matched to sequences using the Sequest^45^ or ProLuCID^46^ algorithm. The peptide mass search tolerance was set to 3 Da for spectra acquired on the LTQ instrument. Cysteine mass was statically modified by +57.02146 Da to account for sample carboxyamidomethylation; serine, threonine and tyrosine were differentially modified by +79.9663 Da to account for phosphorylation.

### Pharmacological treatment of cortical and hippocampal neurons

Cortical and hippocampal neurons were treated with the ROCK inhibitor Y27632 (20 μM, Calbiochem) for 6 h before fixation. Cortical neurons (14–16 DIV) were treated with Bic (20 μM, Sigma), TTX (1 μM, Aladdin Chemistry) or APV (100 μM, Tocris). Cells were pretreated with anisomycin (40 μM, Sigma) and actinomycin D (10 μM, Sigma) for 1 h before Bic treatment. Ephrin-A1-Fc (5 μg ml^−1^, R&D Systems) and Fc (Jackson Immunoresearch Labs) were preclustered with goat or mouse antibody to human Fc in a ratio of 1:4.5 and incubated at room temperature for 60 min before use. Full-length blots are shown in Supplementary Fig. 7.

### Immunofluorescence staining and real-time quantitative PCR

Cultured neurons were fixed with 4% paraformaldehyde and 4% sucrose in PBS (pH 7.4) for 15–20 min at room temperature. The cells were subsequently washed with Dulbecco's PBS and mounted. Mouse brains were immersed in 4% paraformaldehyde in PBS (pH 7.4) for 36 h. Coronal sections (50 μm) were created using a vibrating microtome (Leica) and stained with the indicated antibodies as described previously^47^. *In situ* hybridization was performed as described previously^48^. The sense and antisense RNA probes for CaMKv were synthesized and hybridized in cultured hippocampal neurons overnight at 50 °C. CaMKv mRNA was detected using the Tyramide Signal Amplification kit (Invitrogen). Following the hybridization and tyramide signal amplification detection, neurons were stained with MAP2 antibody.

Total RNA was extracted using the QIAGEN RNA extraction kit according to the manufacturer's instruction. The real-time quantitative PCR was performed with fast-standard SYBR Green Dye using AB7500 real-time PCR machine. The following primers were used for real-time PCR: *camkv* mRNA forward 5′-gtgcacaggaacctcaagc-3′, reverse 5′-tcgctgatgacaatctttgag-3′; *hprt1* mRNA (endogenous control) forward 5′-tgacactggtaaaacaatgca-3′, reverse 5′-ggtccttttcaccagcaagct-3′.

### Synaptoneurosome and metabolic labelling

Synaptoneurosomes were prepared from P15–20 mice as described previously^49^. Cortices and hippocampi were dissected and homogenized in ice-cold oxygenated homogenization buffer (125 mM NaCl, 1.2 mM MgSO_4_, 2.5 mM CaCl_2_, 1.53 KH_2_PO_4_, 212.7 mM glucose and 4 mM NaHCO_3_ (pH 7.4)) supplemented with Protease Inhibitor Cocktail^50^ and 40 U ml^−1^ RNase inhibitor (Invitrogen). After centrifugation for 2 min at 2,000*g*, the supernatant was collected and passed through 100- and 10-μm Millipore filters. The flow-through was centrifuged for 15 min at 1,000*g* to collect synaptoneurosomes. The newly synthesized proteins were labelled with the Click-iT (Invitrogen) as described previously^51^. The synaptoneurosome was treated with cycloheximide (60 μM, Calbiochem) for 15 min after 10 min of recovery at 37 °C. Full-length blots are shown in Supplementary Fig. 7.

### GTPase activation assay

RhoA GTPase activity was measured by pull-down analysis. In brief, cultured cortical neurons were washed twice with ice-cold Tris-buffered saline and lysed with lysis buffer (50 mM Tris (pH 7.2), 1% Triton X-100, 0.5% sodium deoxycholate, 0.1% SDS, 500 mM NaCl, 10 mM MgCl_2_, 1 mM PMSF, and 10 μg ml^−1^ leupeptin and aprotinin). Lysates were incubated with agarose beads conjugated with GST-RBD, a Rhotekin domain that specifically binds the GTP-bound form of RhoA, on ice for 90 min. The beads were washed four times with Tris buffer containing 1% Triton X-100, 150 mM NaCl, 10 mM MgCl_2_, 1 mM PMSF, and 10 μg ml^−1^ leupeptin and aprotinin. The beads were resuspended with sample buffer, and bound proteins were separated by SDS–polyacrylamide gel electrophoresis. The active GTP-bound RhoA was detected using RhoA antibody. Full-length blots are shown in Supplementary Fig. 7.

### Electrophysiology

Whole-cell recordings were obtained from hippocampal neurons at 18–19 DIV using the MultiClamp 700A amplifier (Axon Instruments). The pipettes used typically had a resistance of 3–5 MΩ when filled with an internal solution comprising 130 mM K^+^ gluconate, 10 mM KCl, 10 mM HEPES, 1 mM EGTA, 2 mM MgCl_2_, 2 mM Na_2_ATP and 0.4 mM Tris GTP (pH adjusted to 7.3). The cells were continuously superfused with an external solution comprising 125 mM NaCl, 4.0 mM KCl, 1.2 mM MgSO_4_, 2.5 mM CaCl_2_, 1.2 mM KH_2_PO_4_, 11 mM glucose and 26 mM NaHCO_3_ at a flow rate of 1.5–2.0 ml min^−1^. The external solution was bubbled with carbogen and maintained at 34±1 °C. TTX (1 μM) and Bic (10 μM) were included to block action potentials and GABA transmission, respectively. Once a whole-cell recording was achieved, the cell was held at −70 mV to record the mEPSCs and filtered at 3 kHz for 5–15 min. AMPA receptor involvement in mediating the mEPSCs was confirmed by the addition of CNQX (20 μM) that eliminated all events. The mEPSCs were analysed by MiniAnalysis (Synaptosoft).

For slice recording, mice were decapitated and brains were immediately removed and immersed in ice-cold artificial cerebrospinal fluid (125 mM NaCl, 2.0 mM KCl, 1.2 mM MgSO_4_, 2.5 mM CaCl_2_, 1.2 mM KH_2_PO_4_, 11 mM glucose and 26 mM NaHCO_3_), which was continuously bubbled with 95% O_2_ and 5% CO_2_. Parasagittal sections (300 μm) were cut using a vibrating microtome (Leica). Slices were preincubated in oxygenated artificial cerebrospinal fluid at 34±1 °C for at least 2 h.

A planar multi-electrode recording set-up (MED64 system, Alpha Med Sciences) was used to record the fEPSPs. Each slice was superfused with 100 ml oxygenated artificial cerebrospinal fluid, which was recirculated at a flow rate of 1.0–1.5 ml min^−1^. The fEPSPs were recorded from the dendritic layer of CA1 neurons by choosing an electrode in the Schaffer collateral pathway as the stimulating electrode. The input–output curve was generated by delivering electrical stimuli from 10–100 μA, and the peak amplitude of the fEPSPs was measured. A stimulation intensity that evoked the fEPSP with a magnitude of 30–40% of the maximum response was chosen. After allowing a stable baseline of 30 min, an induction protocol that evoked L-LTP was applied; five spaced trains (inter-train interval of 5 min) were applied, each consisting of six theta bursts (100 Hz, inter-burst interval of 300 ms)^23^. The field potential was monitored for at least 3 h after the conditioning stimuli. The L-LTP magnitude was quantified as the percentage change in the average amplitude of the fEPSP taken from the 200- to 210-min interval after L-LTP induction compared with that of the baseline average. NMDA receptor long-term depression was induced by low-frequency stimulation (1 Hz) for 15 min. All electrophysiology experiments were performed and analysed in a blinded manner.

### Virus injection and osmotic pump implantation

Virus injection was performed using 2–3-month-old adult C57/B6 mice. Vesicular stomatitis virus glycoprotein G-pseudotyped lentiviral particles with pFUGW control or pFUGW-CaMKv shRNA (shCaMKv) were delivered bilaterally into the hippocampal CA1 region (anteroposterior, −2.00 mm; mediolateral, ±1.70 mm; dorsoventral, −1.4 mm; relative to the bregma), and the mice were returned to their home cages for 4 weeks before analysis. Lentivirus was injected using an animal stereotactic apparatus (Stoelting) and the quintessential stereotaxic injector (Stoelting) with an injection volume of 3 μl at 0.10 μl/min (virus titre: 2 × 10^8^ μl^−1^). To analyse spine density, the viruses were diluted 10-fold with PBS before injection to label isolated CA1 neurons.

The micro-osmotic pumps (1003D, Alzet) were implanted 3 days before the perfusion following manufacturer's instruction. ROCK inhibitor Y27632 was diluted in PBS as described.

### Image acquisition and quantitative analysis

Images cultured neurons were acquired by a Nikon A1 confocal microscope with a × 60 oil-immersion objective. We collected 8–12 serial individual optical sections (*Z*-interval of 0.5 μm). Images from the same experiment were obtained using identical acquisition settings, and image analysis was performed using Metamorph software (Meta Image Series 7.5, Universal Imaging). For quantification, three dendritic segments from each cultured neuron were analysed, and the head width, neck width and length of individual protrusions on the dendritic shaft were measured. If the head/neck width ratio exceeded 1.5, the protrusion was defined as a dendritic spine. For brain slices, images were acquired by a Leica TCS SP8 confocal microscope. The proximal secondary dendrites from the apical shaft of CA1 pyramidal neurons were selected, and spine morphology and density were analysed as described above. All data are presented as the mean±s.e.m. from at least three independent experiments.

### Animals and behavioural studies

The transgenic mice and C57BL/6J mice were produced by the Animal Care Facility of The Hong Kong University of Science and Technology (HKUST), and the experiments were approved by the HKUST Animal Ethics Committee and conducted in accordance with the Code of Practice Care and Use of Animals for Experimental Purposes of Hong Kong.

The MWM was performed using 3–4-month-old male C57BL/6J mice injected with shCaMKv or scrambled (Scr) lentivirus 1 month prior. The MWM was performed in a circular tank (diameter: 1.5 m) filled with opaque water. The hidden platform location was alternated for each cage (*n*=4 mice). Release points were randomized for each trial. The swimming path of the mice was recorded by video camera and tracked and analysed using Ethovision XT (Noldus Software). In each trial, each mouse was allowed to search for the platform for a maximum of 90 s. Once the mouse found the platform and remained for 5 s, it was left to dry and returned to its home cage. On the first day, the mice that did not locate the platform during the 90 s were gently guided to the platform and left on it for 5 s. Three to four training trials were conducted each day for 7 days, and the latency for each trial was recorded. On day 8, the probe trial was performed; each mouse was allowed to swim for 60 s with the platform removed. Performance was assessed according to the time spent in each quadrant and the cumulative distance travelled to where the hidden platform was originally located.

The open-field test was performed in an open-top opaque polyethylene box (40 × 40 × 40 cm), and locomotion was tracked and analysed using Ethovision XT (Noldus Software). Each mouse was placed in the centre of the box and allowed to explore the arena for 20 min in dim light conditions. Horizontal spontaneous locomotor activity was monitored in clean home cages for 2.5 h in the dark. Mice were individually placed in standard cages located in chambers equipped with a computer-monitored infrared photobeam system (Photobeam Analysis Software, San Diego Instruments). Locomotor activity was measured as sequential adjacent beam breaks. T-maze spontaneous alternation task was performed as described^52^. Two trials were given to mice in quick succession. On the first trial, mice were started from the base of T-maze and allowed to choose one of the arms; on the second trial, mice were allowed to choose the arms without the central partition in the maze. Each mouse was tested six times within 1 day, and the duration and destination were recorded manually. All behavioural experiments and analyses were performed with the experimenter blinded to the treatment groups.

### Data availability

The data that support the findings of this study are available from the corresponding author on request.

## Additional information

**How to cite this article:** Liang, Z. *et al*. The pseudokinase CaMKv is required for the activity-dependent maintenance of dendritic spines. *Nat. Commun.* **7,** 13282 doi: 10.1038/ncomms13282 (2016).

**Publisher's note:** Springer Nature remains neutral with regard to jurisdictional claims in published maps and institutional affiliations.

## Supplementary Material

Supplementary Information Supplementary Figures 1 - 7 and Supplementary Table 1
