# Supplementary material for: The pseudokinase CaMKv is required for the activity-dependent maintenance of dendritic spines
Source: Nat Commun. 2016 Oct 31;7:13282. doi: 10.1038/ncomms13282 (PMC5095516; doi:10.1038/ncomms13282)
Supplement: Supplementary Information — Supplementary Figures 1 - 7 and Supplementary Table 1 [file ncomms13282-s1.pdf]

Supplementary Figures

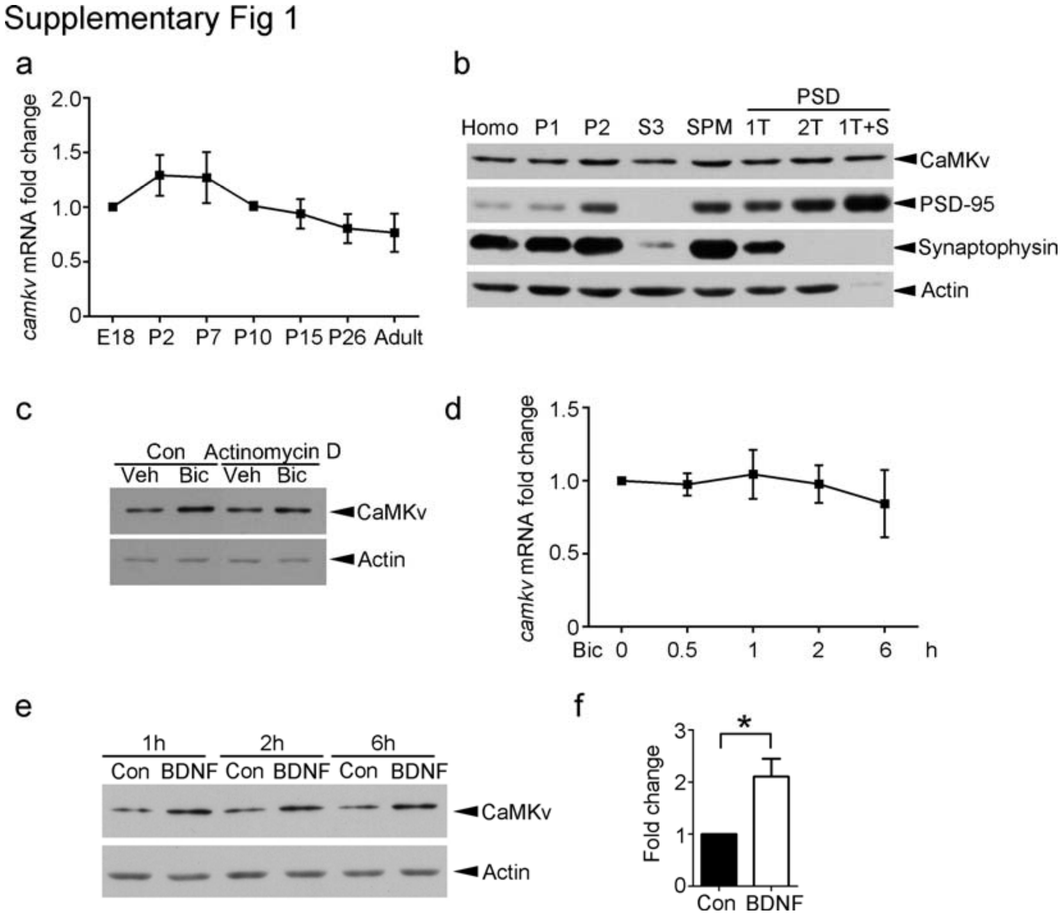

Supplementary Figure 1. CaMKv protein synthesis at synapses is regulated by neuronal activity. (a) Real-time qPCR analysis of *camkv* mRNA level in the mouse forebrain at the indicated developmental stages (n = 3 independent experiments, normalized to *hprt1* mRNA). (b) CaMKv was present in the synaptic plasma membrane (SPM) and postsynaptic density (PSD) fractions of adult rat brains. (c) Induction of CaMKv expression by bicuculline (Bic) is not affected by gene transcription inhibition by actinomycin D. Cortical neurons at 14 days *in vitro* (DIV) were pretreated with actinomycin D (10 μM) for 1 h and incubated with bicuculline (40 μM) for 1 h. (d) Real-time qPCR analysis of *camkv* mRNA level with bicuculline

(Bic) treatment for indicated duration ($n = 3$ independent experiments, normalized to *hprt1* mRNA). (e) CaMKv expression was increased after BDNF treatment. (f) Cortical neurons at 14 days *in vitro* (DIV) were treated with BDNF (100 ng/ml) for 1 h ($n = 3$ independent experiments, $*p < 0.05$, Student's *t*-test).

Supplementary Fig 2

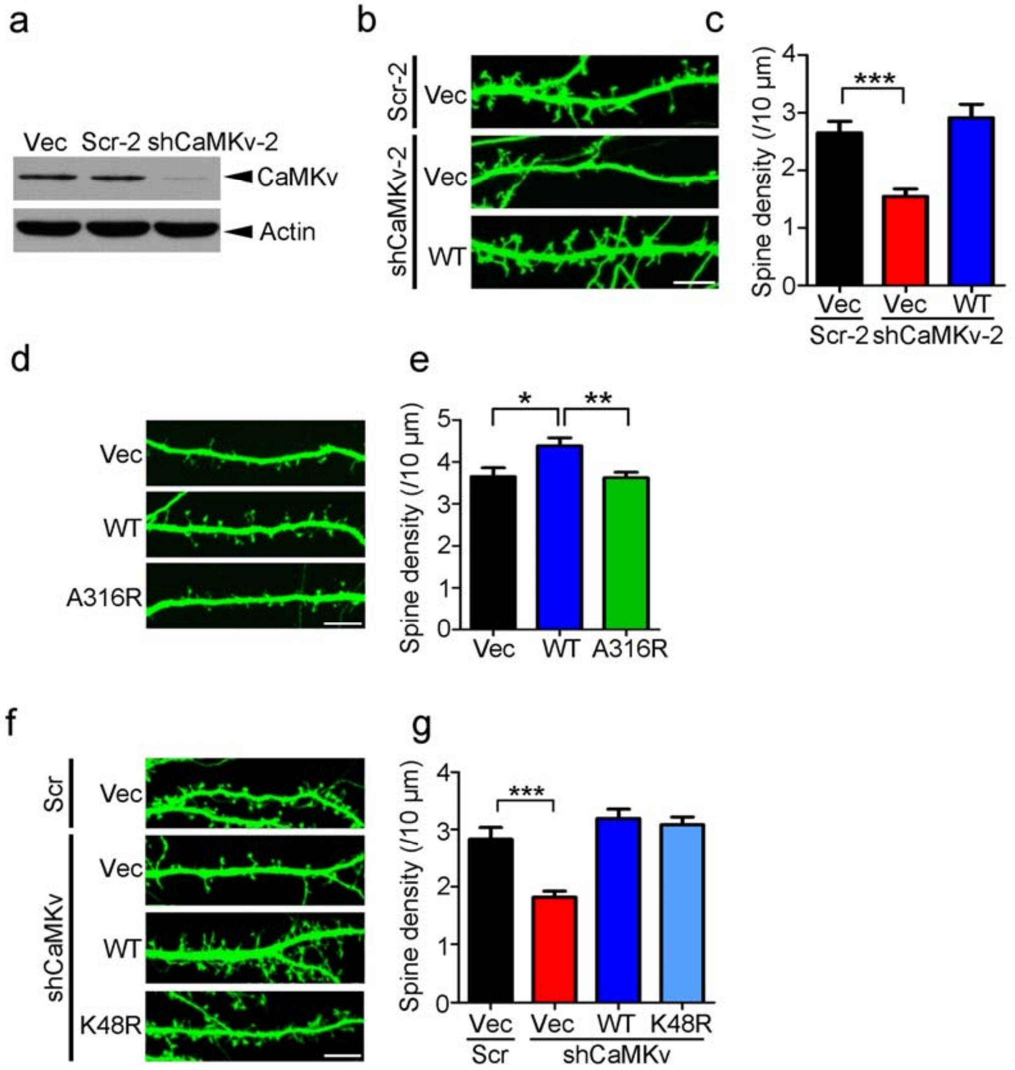

Supplementary Figure 2. CaMKv is required for dendritic spine maintenance. (a) Primary cortical neurons were transfected with CaMKv shRNA-2 (shCaMKv-2) or the corresponding scrambled control (Scr-2) by nucleofection at 0 DIV. CaMKv expression was drastically reduced by shCaMKv-2 at 5 DIV. (b, c) CaMKv knockdown by shCaMKv-2 significantly decreased dendritic spine density. Spine loss could be rescued by co-expressing the RNAi-resistant wild-type (WT) CaMKv (scale bar: 10 µm; Scr-2: n = 18 dendrites, shCaMKv-2: n = 15 dendrites, rescue: n = 21 dendrites, ***$p < 0.001$, one-way ANOVA, Tukey's multiple comparison test). (d, e)

Overexpression of WT CaMKv but not the CaMKv A316R mutant significantly increased spine density (scale bar: 10 μm; $n = 42$ dendrites per condition, $^{*}p < 0.05$, $^{**}p < 0.01$, one-way ANOVA, Tukey's multiple comparison test). (f, g) CaMKv kinase activity is not required for spine maintenance. CaMKv knockdown significantly decreased dendritic spine density. Spine loss could be rescued by co-expressing the RNAi-resistant WT CaMKv or the CaMKv K48R mutant (scale bar: 10 μm; Scr: $n = 15$ dendrites, other conditions: $n = 21$ dendrites, $^{***}p < 0.001$, one-way ANOVA, Tukey's multiple comparison test).

Supplementary Fig 3

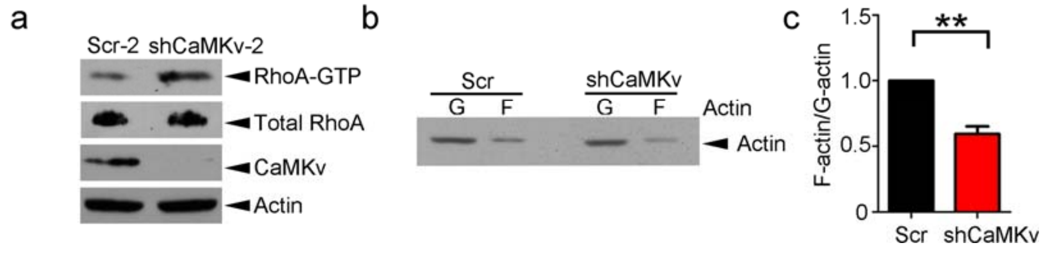

Supplementary Figure 3. CaMKv regulates spine morphogenesis via RhoA inhibition. (a) shCaMKv-2 was expressed in cultured cortical neurons by nucleofection, and active RhoA was examined by GTPase assay as described in the Methods. Active RhoA increased after CaMKv knockdown. (b, c) CaMKv regulates actin assembly. Cortical neurons were transfected with shRNA by nucleofection at 0 DIV, and the F-actin/G-actin ratio of CaMKv was examined at 5 DIV. The F-actin/G-actin ratio decreased after CaMKv knockdown (n = 3 independent experiments, $**p < 0.05$, Student's t-test).

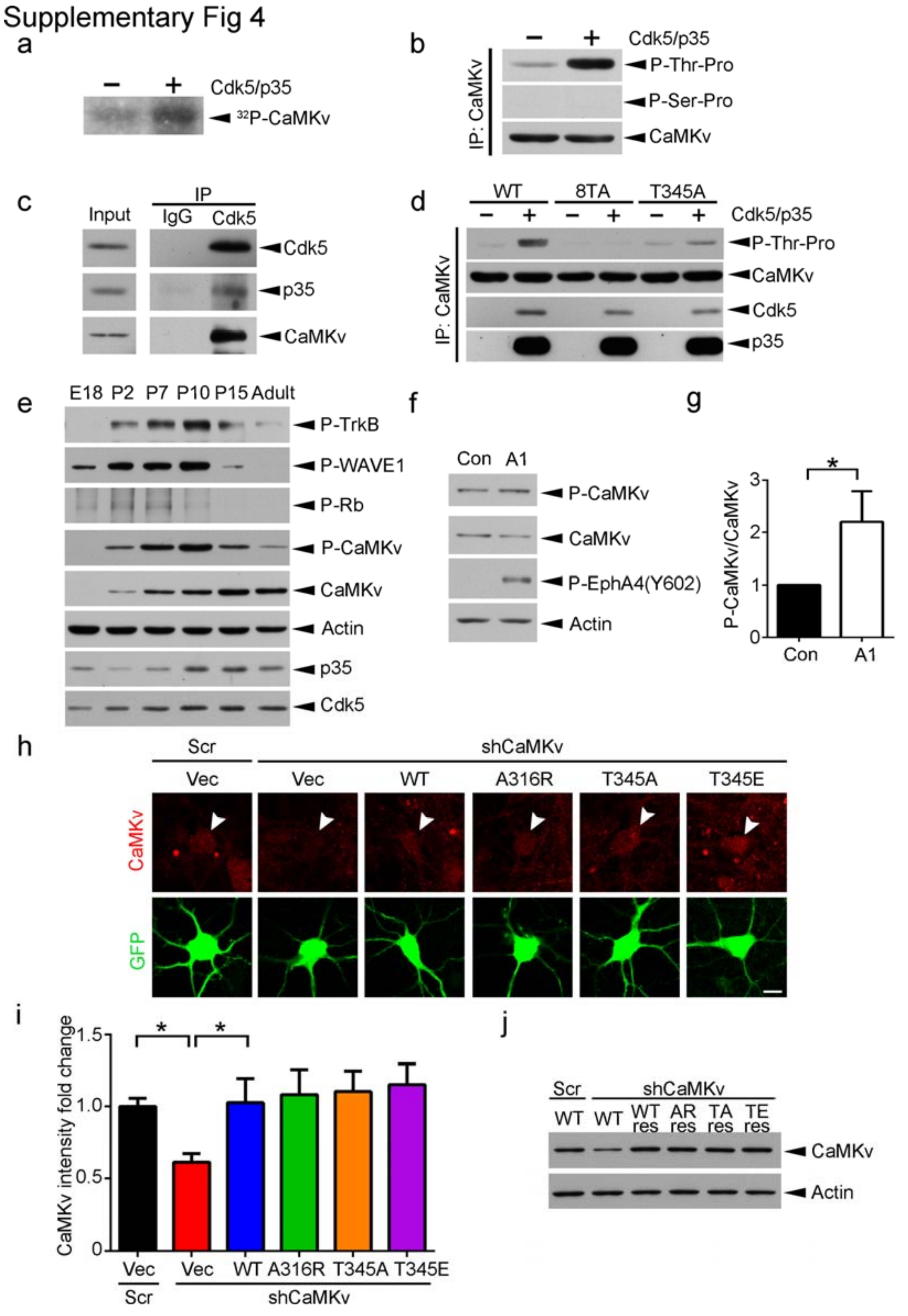

Supplementary Figure 4. Cdk5 phosphorylates CaMKv at Thr-345. (a) CaMKv was phosphorylated by recombinant Cdk5/p35 *in vitro*. CaMKv was overexpressed

and immunoprecipitated from HEK293T cells. The ^{32}P radioactive signal was detected at the corresponding molecular weight (~72 kDa) of CaMKv. (b) CaMKv was phosphorylated by Cdk5/p35 at Thr/Pro site(s). FLAG-tagged CaMKv, Cdk5, and p35 were co-expressed in HEK293T cells, and CaMKv was immunoprecipitated by anti-FLAG antibody, followed by western blotting with proline-directed phospho-threonine or proline-directed phospho-serine antibody. (c) CaMKv was co-immunoprecipitated with Cdk5 and p35 from the mouse brain synaptosome. (d) CaMKv was mainly phosphorylated by Cdk5/p35 at Thr-345. FLAG-tagged WT, the phospho-deficient CaMKv mutant lacking phosphorylation of all eight threonine residues (8TA), or the Thr-345 phospho-deficient mutant (T345A) was co-expressed with Cdk5 and p35 in HEK293T cells. After immunoprecipitation by an anti-FLAG antibody, the phosphorylation state was determined by a proline-directed phospho-threonine antibody. (e) The phosphorylation level of Cdk5 substrates in the mouse forebrain at different developmental stages. (f, g) EphrinA1 treatment (5 μg/ml) for 5 min increases the phosphorylation of CaMKv. (n = 3 independent experiments, $*p < 0.05$, Student's t-test) (h, i) The expression of CaMKv or its mutants was indicated by the specific antibody against CaMKv. (scale bar: 10 μm; n = 10-12 neurons per condition, $*p < 0.05$, one-way ANOVA, Bonferroni's multiple comparison test) (j) Western blot analysis of the expression of WT, RNAi-resistant (res) WT, the A316R mutant, the T345A mutant, and the phospho-mimetic mutant of CaMKv in HEK293T cells.

Supplementary Fig 5

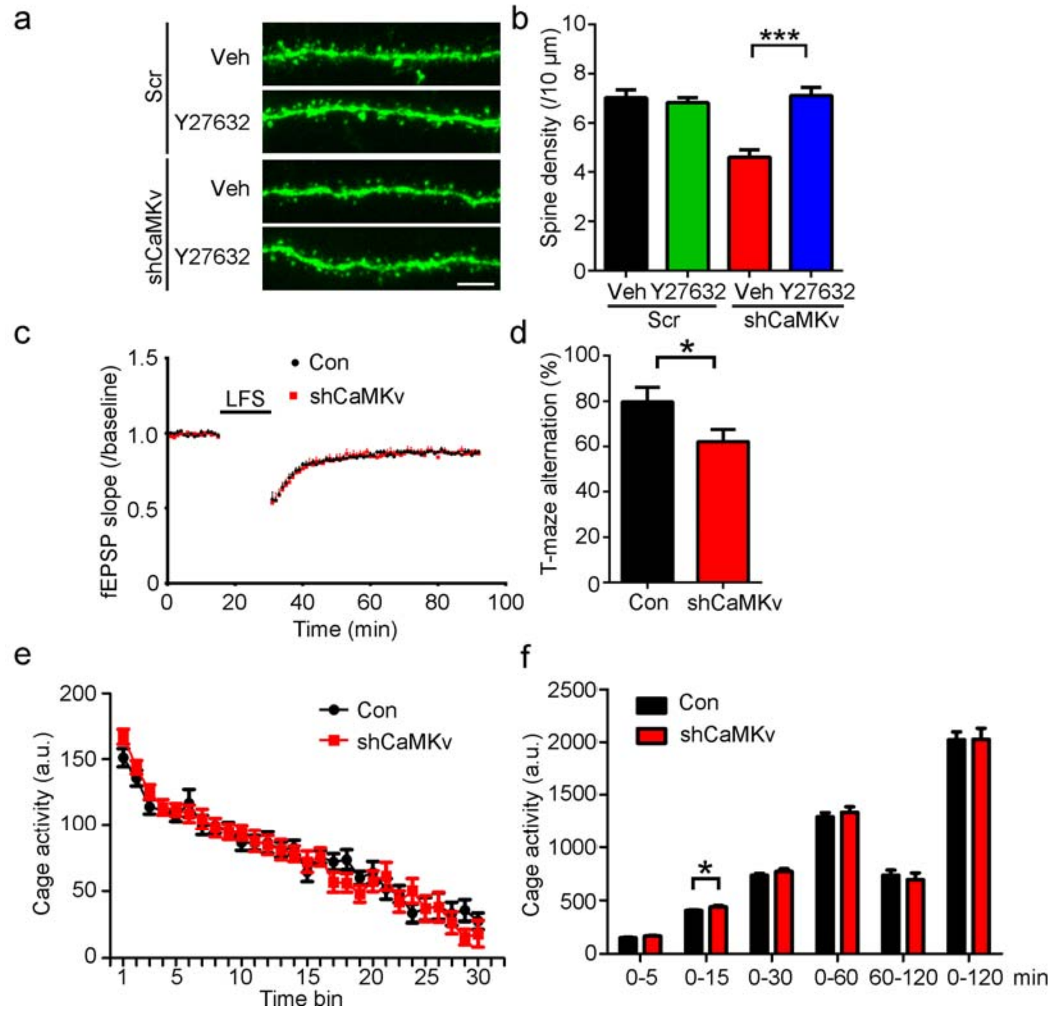

Supplementary Figure 5. CaMKv knockdown in hippocampal CA1 neurons leads to hyperactivity. (a, b) Delivery of ROCK inhibitor *in vivo* restores the decreased spine density after the knockdown of CaMKv. Hippocampal CA1 pyramidal neurons infected with CaMKv shRNA or Scr were treated with the ROCK inhibitor Y27632 (20 μM) or vehicle (Veh) by osmotic pump for 3 days. Y27632 abolished the spine loss induced by CaMKv shRNA lentivirus (scale bar: 10 μm; n = 21-24 dendrites per condition, ***$p < 0.001$, one-way ANOVA, Tukey's multiple comparison test). (c) CaMKv knockdown by shCaMKv did not affect NMDA receptor long-term depression. (Con: n = 3 mice, shCaMKv: n = 3 mice). (d) CaMKv

knockdown mice exhibited decreased alternation rate in a T-maze spontaneous alternation task (Con: $n = 14$ mice, shCaMKv: $n = 15$ mice, $*p < 0.05$, Student's t-test). (e) Home cage locomotor activity in CaMKv knockdown and control mice (the data are represented in 5-min time bins). (f) CaMKv knockdown mice exhibited significantly increased locomotor activity during the initial 15 min (Con: $n = 16$ mice, shCaMKv: $n = 19$ mice, $*p < 0.05$, Student's t-test).

Supplementary Fig 6

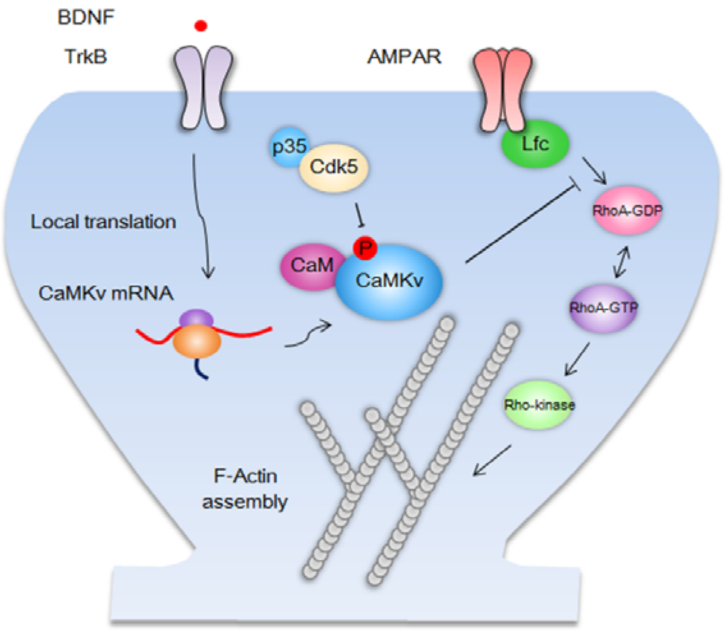

Supplementary Figure 6. Schematic diagram illustrating the proposed function of CaMKv in the regulation of activity-dependent spine morphogenesis

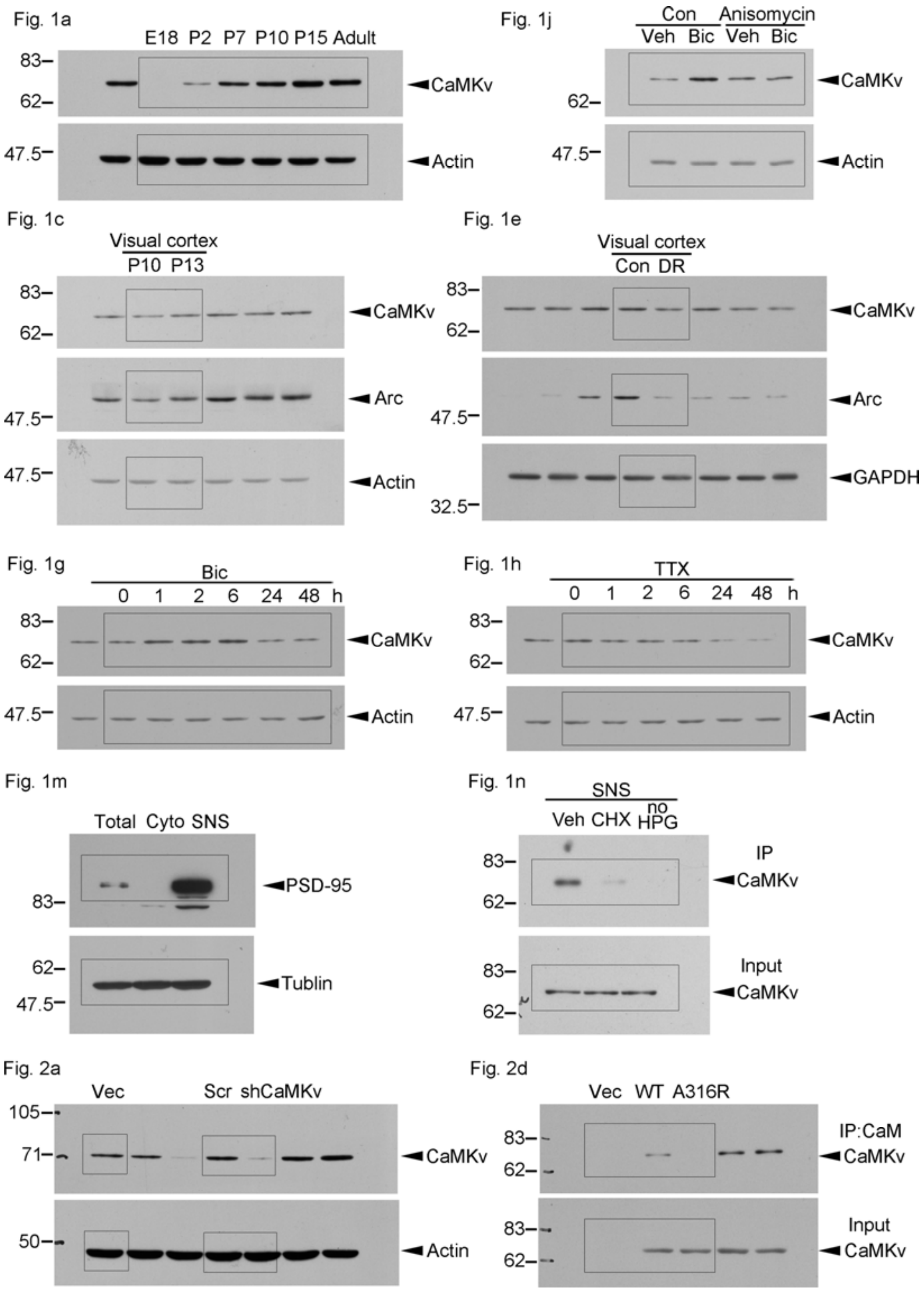

Supplementary Figure 7 Images of full-length blots

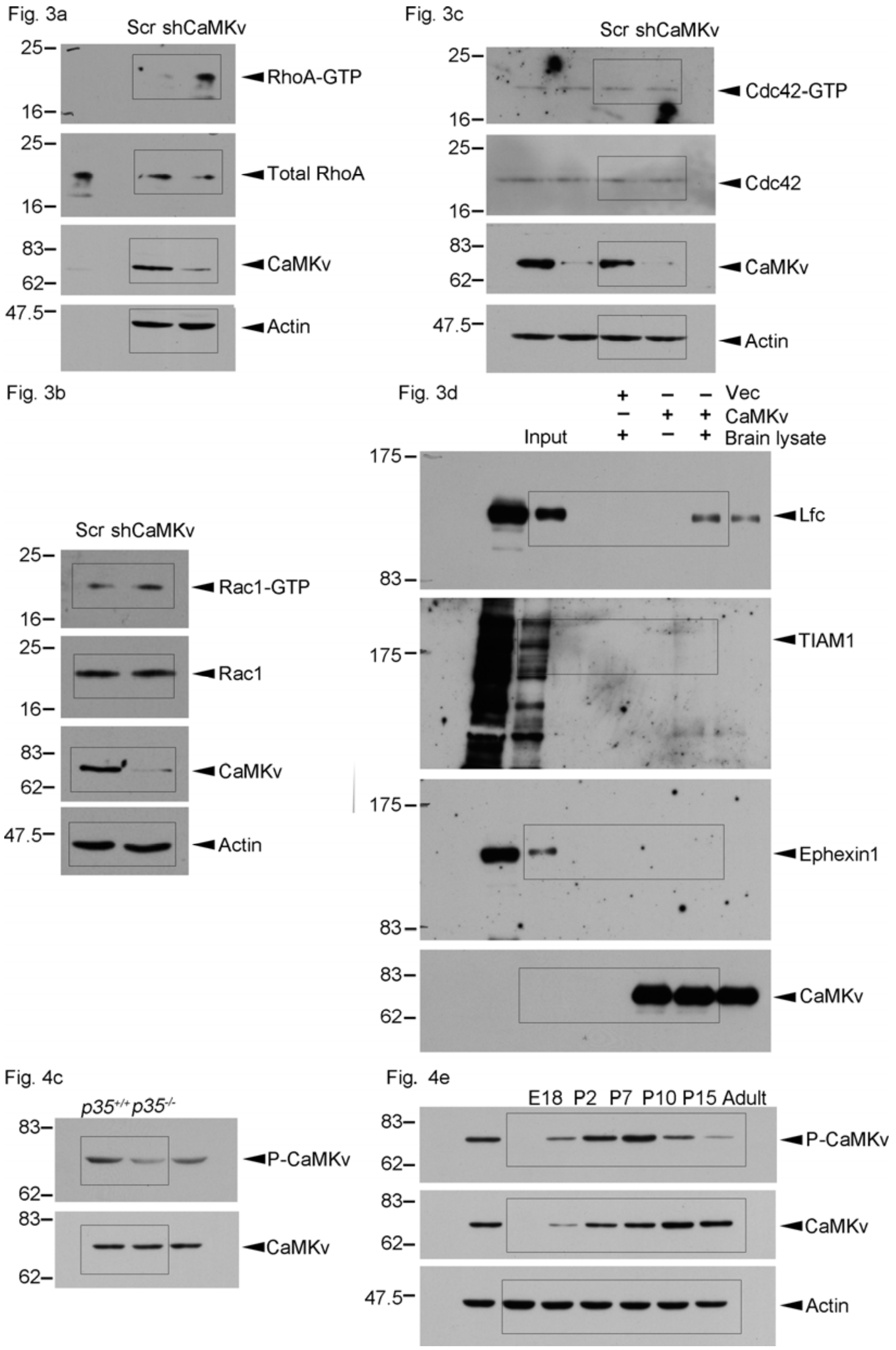

Supplementary Figure 7 (continued) Images of full-length blots

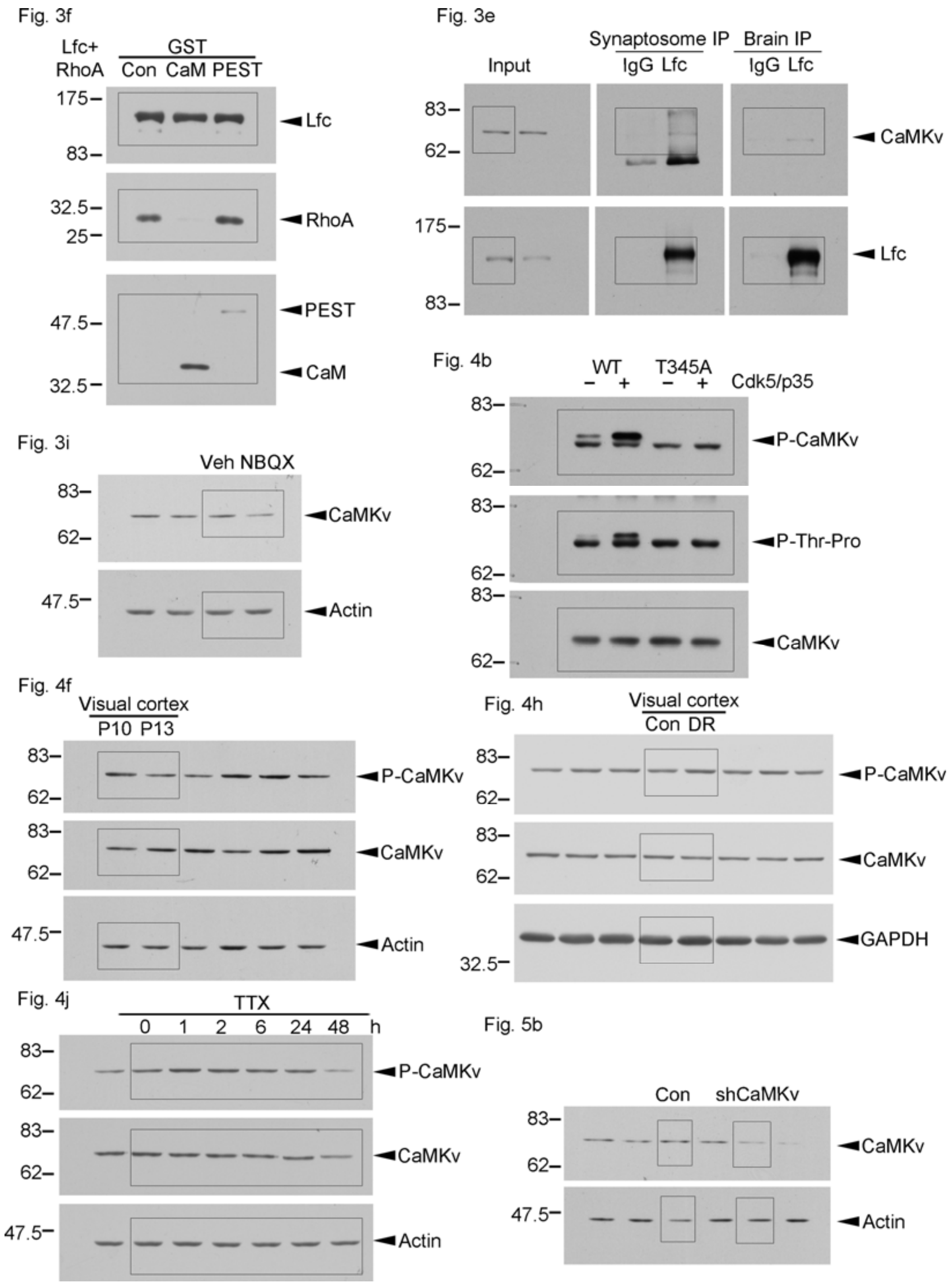

Supplementary Figure 7 (continued) Images of full-length blots

Supplementary Table 1: primers for the CaMKv mutants

Constructs	Primers
shCaMKv<br>RNAi-resistant	Forward 5′-gcggaaggcagccaaaaatgaaattggaatcctcaagatg-3′<br>Reverse 5′-catcttgaggattccaatttcatttttggctgccttccgc-3′
shCaMKv-2<br>RNAi-resistant	Forward 5′-caacaggctgaagaactccaaaatagtcatcagcgactttcac-3′<br>Reverse 5′-gtgaaagtcgctgatgactattttggagttcttcagcctgttg-3′
CaMKv A316R	Forward 5′-gagccaagtggaagaagcgtgtccgagtgaccac-3′<br>Reverse 5′-gtggtcactcggacacgcttcttccacttggctc-3′
CaMKv T345A	Forward 5′-gcaaaggcagcagccgcccctgagccg-3′<br>Reverse 5′-ccagcagccccaggagcggcagcgtctgaagc-3′
CaMKv T345E	Forward 5′-gcttcagacgctgccgaacctggggctgctgg-3′<br>Reverse 5′-ccagcagccccaggttcggcagcgtctgaagc-3′
